# Improving Patient Race and Ethnicity Data Capture to Address Health Disparities: A Case Study From a Large Urban Health System

**DOI:** 10.7759/cureus.20973

**Published:** 2022-01-05

**Authors:** Ruben D Vega Perez, Lyndia Hayden, Jefri Mesa, Nina Bickell, Pamela Abner, Lynne D Richardson, Ka Ming Ngai

**Affiliations:** 1 Emergency Medicine, Icahn School of Medicine at Mount Sinai, New York City, USA; 2 Office for Diversity and Inclusion, Icahn School of Medicine at Mount Sinai, New York City, USA; 3 Medicine, Icahn School of Medicine at Mount Sinai, New York City, USA

**Keywords:** feasibility of implementing system-wide interventions, project demonstrates, health equity, data quality, capturing accurate race and ethnicity data

## Abstract

While research and efforts to promote health equity abound, the persistence of disparities by race and ethnicity underscores the limitations of fragmented interventions and the need for systematic, multipronged approaches to health equity. The foundational step towards reducing health disparities is the establishment of the basic information needed to identify and measure those differences, i.e., the accurate capture of race and ethnicity information of all patients. To that end, we present a case study outlining a multifaceted approach for improving the capture of race and ethnicity data in an outpatient setting culminating in a 76% improvement in the completeness of this information. The effectiveness of this plan and its scalability within a large urban health system may benefit similar institutions seeking to improve the collection of race and ethnicity information and the reliability of their data. To this aim, we present an approach relying on the assessment and evaluation of system needs, modification of data infrastructure to align with goals, training, and education of relevant stakeholders, implementation and responsive action to results, and acknowledging limitations and lessons learned. We emphasize that cross-departmental collaboration, stakeholder engagement, institutional support, and culture of anti-racism were essential to the success of this initiative.

## Introduction

The coronavirus disease 2019 (COVID-19) pandemic’s disproportionate burden on communities of color and the clamors for racial justice engulfing the nation make evident [1,2]. One of the most pressing tasks confronting health care systems today centers on eliminating health care disparities and providing equitable care. Almost two decades after the publication of the seminal Institute of Medicine Report, "Unequal Treatment,” health systems continue to grapple with ensuring equitable care for patients of all races and ethnicities [3,4]. Since then, significant advancements have developed in the recognition that effective interventions to improve patient care equitably require reliable demographic data and accurate measurements of health disparities. According to the Agency for Health Care Research and Quality, an essential step towards the goal of equitable care is systematically identifying patient populations, addressing the needs of these populations, and monitoring improvements over time [5]. This process largely depends on the collection of granular demographic data like race, ethnicity, and language preference (REL) facilitating the stratification of quality measures at a level of detail that can identify variation in health and health care among vulnerable groups [6]. The challenge lies in identifying the best practices for the systematic and reliable identification of patient demographics.

We will describe the process in ensuring race/ethnicity capturing in one of the largest health institutions in New York City providing care for over 150,000 inpatient admissions and four million outpatient visits every year [7]. The Office for Diversity and Inclusion (ODI) within the health system is dedicated to promoting and addressing diversity, equity, and inclusion as it relates to health care. ODI leads the development and implementation process for the collection of patient race and ethnicity data in a large and complex health system to support quality research in health equity, identify and address health care disparities, and improve health outcomes for all. New York’s Statewide Planning and Research Cooperative System (SPARCS) has mandatory requirements for race and ethnicity data collection. Yet, these data fields were often found by health service researchers to have missing or inaccurate data. ODI works with the health system to ensure accurate collection of race and ethnicity data fields as required by SPARCS [8]. Prior to this initiative, our health system did not systematically collect this demographic data. Rather, it passively requested patients to report race and ethnicity. One way to improve such data is organizational reform to recognize that inaccurate race and ethnicity data can contribute to health disparities. Given the fundamental need for reliable REL data for the tracking of health equity, ODI identified this as a priority, beginning with ethnicity and race data. This initiative also recognizes that addressing disparities will likely soon become a key component of The Joint Commission’s accreditation standards, the National Quality Forum’s quality measures, pay-for-performance contracts, and community benefit principles that are now under close federal scrutiny [9]. Here, we present policy experience from the implementation of a multi-year patient registration data collection improvement process (PRDCIP) as a case study on the systematic expansion of REL data capture in a service line within a large urban health system.

## Materials and methods

This study was approved by the institutional review board of Icahn School of Medicine at Mount Sinai as exempt from human research as defined by DHHS regulations (45 CFR 46.104 {d}). The project consisted of five phases outlining a systematic overhaul of the procedures for the collection of race and ethnicity data as (1) assessment and evaluation, (2) infrastructure modification, (3) training and education, (4) implementation and response to results, and (5) acknowledging limitations and lessons learned. Internal Medicine Associates (IMA) is a primary care setting located in East Harlem, New York, chosen as the pilot site for PRDCIP.

**Figure 1:**
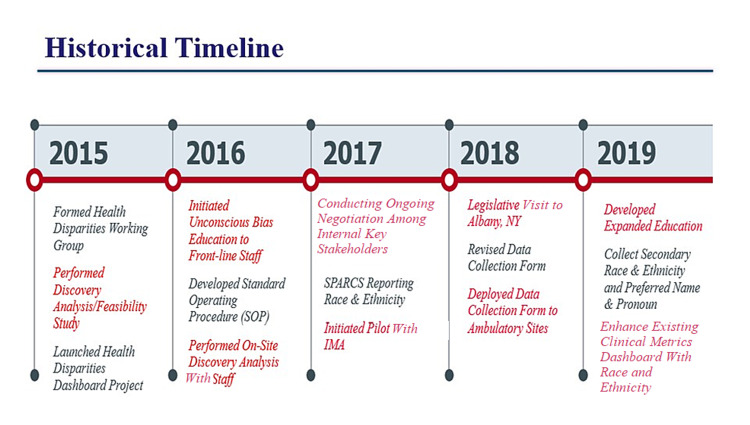
Multi-year timeline for the initiative.

Beyond reporting compliance, the goals of this initiative stressed the importance of capturing accurate race and ethnicity data self-reported by the patient by educating and training staff on the significance of collecting this data to help vulnerable and underserved populations, with a long-term goal of >90% patient self-identification in race and ethnicity. In addition, unconscious bias training was facilitated by ODI for staff involved in the project. This was stressed as an essential step towards establishing equity as a collective priority and aligning stakeholders’ understanding of their roles in reducing disparities. Figure 1 illustrates the multi-year timeline for this initiative, which demonstrates the extensive planning and preliminary actions that were required to set the foundation of the program.

**Figure 2:**
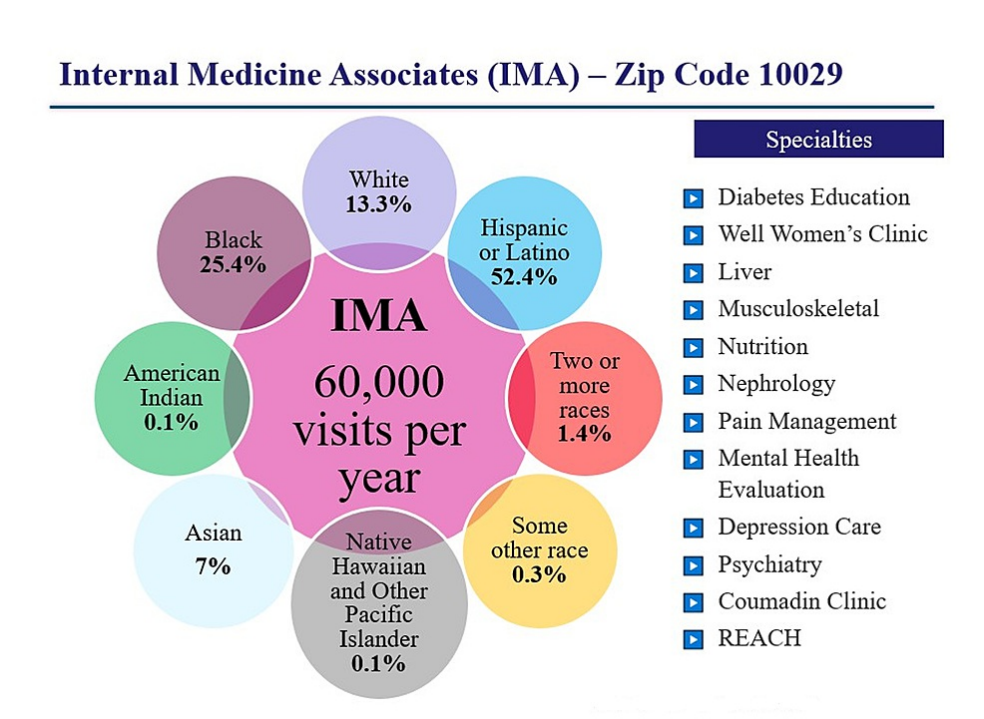
Specialty and racial-ethnic makeup of Internal Medicine Associates (IMA), located in zip code 10029. REACH Program: Respectful and Equitable Access to Comprehensive Healthcare Program

IMA is a hospital-based ambulatory unit staffed by our system physicians. In 2016, this unit was selected to spearhead our health system’s race and ethnicity data improvement initiative because of the range of specialties offered and the diversity of its patient population (for IMA specialty and racial/ethnic makeup in 2016) (Figure 2).

While most U.S. hospitals collect data on patients' race or ethnic group in some form, methods and data quality vary, and very few record this information systematically [10]. The opportunity for hospital race and ethnicity data capture is also limited by the fact that most patients tend to visit the hospital only a few times per year, if at all [10]. As an outpatient primary care site, IMA presented an important access point for systematic demographic data collection for the larger hospital system given its more frequent encounter with patients and wider community reach.

## Results

The purpose of this pilot project (the patient registration data collection improvement process) was to identify race and ethnicity data deficits, establish a framework through which disparities could be identified and monitored, and comply with state mandates on data collection, including SPARCS. Through a series of systematic interventions coupled with patient and staff empowerment, IMA improved its capture of race and ethnicity data decisively.

Phase I - assess and evaluate

To guide the conceptual framework of this initiative, a health disparities working group was formed in 2015. During that time, a process assessment and a feasibility study identified key staff and existing REL data collection processes, conducted clinic staff interviews, and ultimately determined that the health system had no reliable or cohesive plan for the collection of REL data. Furthermore, these system evaluations established the need for, and launched, a health disparities dashboard project with the aim of tracking disparities as REL data became more reliable. The tangible product from these foundational efforts was the development of a standard operating procedure document as a guideline for the scope and protocols of this REL data improvement project. Additionally, a conceptual list of essential clinical quality measures was selected to be monitored for potential disparities - high blood pressure, uncontrolled diabetes, mortality, and readmission.

Alongside the aforementioned efforts, ODI performed on-site patient and staff interviews and group discussions at IMA, once the site was selected for the pilot project. Based on the feedback gathered, ODI developed training materials to address identified challenges and barriers and conducted ongoing conversations to monitor progress during later phases. This included patient and front-desk staff misconceptions about best methods for data collection, the need for this data to correct disparities, and the existence of racial and ethnic disparities themselves.

ODI also conducted ongoing dialogue and managed competing interests among internal key stakeholders, including the finance and information technology (IT) departments. Senior leadership from ODI and IMA also made a legislative visit to Albany, NY, to advocate and validate that the race and ethnicity categories selected for the project aligned with SPARCS reporting requirements. This was done in effort to convince hesitant stakeholders who were concerned that any changes to the existing reporting process could jeopardize compliance and/or reimbursements. Based on that visit to Albany and on existing SPARCS demographic categories, a race/ethnicity data collection form functionalizing the goals of the project was developed. All race and ethnicity options, with the exception of descriptions for the Black and African American category, were ultimately sourced from SPARCS.

Phase II - modify system infrastructure

Although our health system utilizes Epic (Verona, WI: Epic Systems Corporation) as the single electronic health record, the health system relies on seven separate core registration systems as a result of hospital mergers and acquisitions since 2013 (there are migration plans in progress to transfer all registration systems to Epic). As a result, all registration system managers and their respective integration services teams were engaged in the effort to create the new race and ethnicity data entry fields, make entries mandatory, and add more descriptive identities as listed in the patient race and ethnicity information collection form beyond the standard categories of American Indian or Alaska Native, Asian, Black, Hispanic, Native Hawaiian or Pacific Islander, or White. It was important to be consistent among all systems beyond IMA because patients frequently visit multiple locations. These efforts would facilitate the future expansion of the IMA pilot project.

Phase III - educate and train

The education and training stage was a key component to the success of the PRDCIP. While phase I established the need for training and a cohesive understanding of the goals and need for PRDCIP among relevant staff, phase III addressed those findings by training staff on the best data collection processes and their relevance to health equity. Furthermore, ODI initiated unconscious bias education for front-line staff and emphasized the role of reliable data to achieve health equity and social justice. It was critical to develop a curriculum that included relatable content that met the dual objective of emphasizing the importance of collecting the data, but also building confidence and empowering the staff to obtain the information. The learning objectives included the definitions for race and ethnicity, the purpose of collecting the data, the proper use of the race/ethnicity data collection form, a review of where the fields were in each registration system, and a frequently asked questions document for both staff and patients. There were multiple media used such as on-demand training and development platform, instructor-led webinars, and in-person classroom settings. In essence, these efforts established the knowledge, technical skills, and organizational culture conducive to the success of PRDCIP.

Phase IV - implement and respond to results

**Figure 3:**
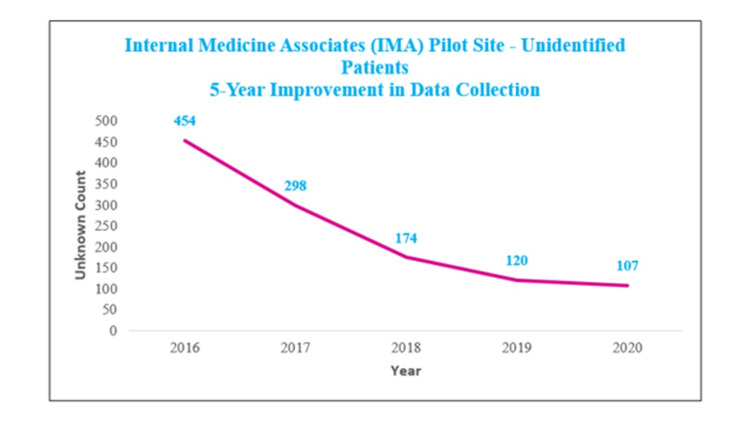
Time series trend of unknown race-ethnicity data fields at IMA over a five-year period. IMA: Internal Medicine Associates

The implementation stage evaluated whether the previous phases and the implementation of the new processes aligned with the aims of the project through qualitative data and the monitoring of the total numbers of patients with unknown race and ethnicity information. This phase centered and encouraged feedback on the effectiveness of the training and information collection procedures in order to adjust and improve any deficiencies. Of note, emphasizing the important role patients play in helping ensure equitable care for all proved especially persuasive. In this process, it became clear that empowering patients and partnering with them for this goal resonated with both staff and patients. These efforts led to a marked and sustained improvement in the race and ethnicity data within the first year, and 90% data capture by the fifth year represents the time series trend of unknown race and ethnicity data fields at IMA over a five-year period since the onset of PRDCIP, decreasing from 454 unidentified patients in 2016 to 107 in 2020; representing a 76% reduction in unknown fields (Figure 3).

Phase V - limitations and lessons learned

As with most projects, the discovery process does not end at implementation. The IMA pilot project and the early stages of data collection were driven by the IT department and ODI. A successful partnership with the IT department not only demonstrated feasibility and scalability but also facilitated the identification of system weaknesses and further necessary improvements beyond the scope of PRDCIP. These include the need for more organizational accountability, leadership, and streamlining reporting methods and reassessment.

One of the first areas of need for improvement at the health system level to surface from this pilot project included the identification of registration and scheduling leaders given the diversity of registration systems used by the health system. At the time of PRDCIP, IT did not have a consistent list of relevant lead personnel at each hospital site or for each registration system. As a result, IT created the registration governance group which meets on a consistent basis to coordinate upcoming work across multiple sites. Nonetheless, these efforts will likely be enhanced as the health system converges into one single electronic medical record integrating patient registration.

Lastly, the collection of more granular race, ethnicity, and other demographic data along with a reassessment of community demographic trends and health care needs was identified as necessary for further improvement of data quality for health equity. Expansion of additional or intersectional patient identity categories, informed by the demographic makeup of our communities was the logical next step for data collection. These include variables capturing gender identity, language preference, housing status, etc. However, these additional components were deferred for later implementation because some would require new data fields and a significant amount of testing and resources, which conflicted with the scheduled implementation dates of the IMA project.

## Discussion

The patient registration data collection improvement process (PRDCIP) went beyond standard recommendations for improved data collection focusing on methodological interventions in the relevant literature, such as improving system workflows, communication strategies, and procedural training [5,6]. The project was innovative in its emphasis on anti-racism and the explicit role of reliable data as a tool for health equity. In this manner, the IMA pilot project poses important lessons for further expansion and for any health system seeking to improve the collection of race and ethnicity data. The hallmarks of this strategy include the assessment and evaluation of system needs, modification of data infrastructure to align with goals, training and education of relevant stakeholders, implementation and response to results, and acknowledging limitations and lessons learned.

Capturing complete race, ethnicity, and other key demographic data is an essential first step in addressing health disparities. Previous literature has addressed the lack of uniformity and systematic capture of REL as key impediments, but less work has focused on the role of structural racism and unconscious bias as barriers to effective data capture and potential solutions [9,10]. While the pivotal Institute of Medicine Report: “Race, Ethnicity, and Language Data: Standardization for Health Care Quality Improvement” makes indirect reference to these issues, the terms “racism” and “racial discrimination” appear only four times in the 189-page document [5]. Similarly concerning is the fact that many health systems fail to recognize that proactive interventions to promote health equity, such as unconscious bias training, can be implemented to help reduce disparities even before stratification by race and ethnicity is possible. In our experience, this strategy not only increased broad support for addressing health disparities but also facilitated the data collection process. Deliberately implementing anti-racism training and system interventions is as important as ever, as recognized in the recent declaration of racism as a serious public health threat by the CDC [11].

Furthermore, the project implemented an educational component to address common misconceptions by staff about the role of race and ethnicity in healthcare. Through these training and discussions, the front-line staff’s role in registering and interviewing patients was emphasized as an important conduit for improved data capture and addressing healthcare disparities. To address confusion about the role of equity in their day-to-day work, we emphasized that equity and the tools needed to ensure it are an important component of quality. It also became clear that understanding of the ways race, racism, and discrimination can make their way to health care differed across the staff. This required clarification that while most health care professionals and hospitals strive to provide the same level of quality of care to all patients, evidence shows this may not be the case [3]. The bottom line is that if you have not looked at your hospital’s quality data stratified by race and ethnicity, you cannot assume that your hospital does not have disparities. Another key point is that treating everyone the same may not be enough to ensure health equity. For example, patients may respond differently when presented with the same information from a clinician. Ensuring the highest quality of care possible to all patients requires understanding and adapting care to the patient’s unique needs and perspectives, which are often influenced by their social and cultural backgrounds. Only then can high-quality care be achieved in a patient-centered manner. 

Addressing common concerns by patients was equally important. This required further training for staff on patient interview and communication strategies to improve patients' self-reported race and ethnicity identities. Emphasizing the role of the patients in helping ensure equitable care for all proved persuasive. This also helped establish rapport. For any strategy requiring personal information like race and ethnicity, it is also essential to address potential hesitance and concerns about possible misuse of this data. Improved collection of this information will allow hospitals and facilities to gain a better understanding of the patients and communities they serve and address the differences in health outcomes they may face. Lastly, one of the most important parts of any strategy like this involves forming partnerships, addressing system barriers, and persuading key stakeholders about the value of health equity.

Identifying early champions and leaders in the implementation of any initiative for health equity is essential. But equally important is the need for advocacy, negotiation, and buy-in from other key stakeholders who can facilitate the success and sustainability of historically neglected initiatives. At our health system, existing resources and support from leadership committed to this work made PRDCIP feasible. Likewise, continued collaboration with regulators and relevant health system stakeholders like the IT department is also important to ensure the durability of these efforts, and alignment of all interests involved.

## Conclusions

PRDCIP demonstrates the feasibility of implementing system-wide interventions to greatly improve the capture of race and ethnicity data essential for health equity. Like any other process in health services, the collection of accurate race and ethnicity data is essential for effective, patient-centered, timely, efficient, and equitable care. Likewise, the health system continues to support an education and awareness campaign by distributing advertising materials on the importance of REL reporting and other demographic data. These initiatives have culminated in a race/ethnicity/language capture rate dashboard which monitors the unknown values for these factors with a goal of >90% patient self-identification. Lastly, and true to the original intent of this work, the improvement of REL data capture has led to the development of core equity dashboards across the health system. These dashboards identify race, ethnicity, age, gender, payors, geographic regions, clinical measures, co-morbidities, and language barriers with the goal of identifying and eventually eliminating disparities in health care. The success thus far and steadfast commitment to these efforts recognize REL data as a powerful tool in the fight to eliminate health injustices.
